# Retraction Note: MircoRNA-33a inhibits epithelial-to-mesenchymal transition and metastasis and could be a prognostic marker in non-small cell lung cancer

**DOI:** 10.1038/s41598-021-88178-8

**Published:** 2021-04-14

**Authors:** Lihua Yang, Jie Yang, Jingqiu Li, Xingkai Shen, Yanping Le, Chengwei Zhou, Shaomin Wang, Shun Zhang, Dazhi Xu, Zhaohui Gong

**Affiliations:** 1 Institute of Biochemistry and Molecular Biology, Ningbo University School of Medicine, Ningbo, 315211 ZJ China; 2 Zhejiang Provincial Key Laboratory of Pathophysiology, Ningbo University School of Medicine, Ningbo, 315211 ZJ China; 3 Department of Chest Surgery, The Affiliated Hospital of Ningbo University School of Medicine, Ningbo, 315020 ZJ China; 4 Department of Oncology, The Affiliated Hospital of Ningbo University School of Medicine, Ningbo, 315020 ZJ China; 5 Clinical Laboratory, Ningbo No. 2 Hospital, Ningbo, 315010 ZJ China; 6 State Key Laboratory of Oncology in South China, Sun Yatsen University Cancer Center, Guangzhou, 510060 GD China; 7 Sun Yatsen University Cancer Center, Guangzhou, 510060 GD China

Retraction of: *Scientific Reports* https://doi.org/10.1038/srep13677, published online 02 September 2015

The Editors have retracted this Article.

After publication, it was brought to the Editors' attention that there were irregularities in a number of figures, specifically:

- Partial overlap of panels between Figures 2G and 4G, and Figures 2H and 4H
- Duplication of parts of panels within Figure 4E and 4F
- Duplication of parts of panels within Figure 2E and 4E, and Figure 4F and 2F

The Editors therefore no longer have confidence in the integrity of the data presented.

Zhaohui Gong disagrees with the retraction of the Article. Lihua Yang, Jie Yang, Jingqiu Li, Xingkai Shen, Yanping Le, Chengwei Zhou, Shaomin Wang, Shun Zhang, and Dazhi Xu did not respond to correspondence in regard to the retraction of this Article.

